# Disclosing ambiguous gene aliases by automatic literature profiling

**DOI:** 10.1186/1471-2164-11-S5-S3

**Published:** 2010-12-22

**Authors:** Roney S Coimbra, Dana E Vanderwall, Guilherme C Oliveira

**Affiliations:** 1 Center for Excellence in Bioinformatics, Research Center René Rachou, FIOCRUZ-MG. Rua Araguari, 741, Barro Preto. Belo Horizonte, MG, Brazil.; 2 Genomics and Computational Biology Group, Research Center René Rachou, FIOCRUZ-MG. Av. Augusto de Lima, 1715, Barro Preto. Belo Horizonte, MG, Brazil.; 3 Molecular Discovery Research, GlaxoSmithKline Moore Dr, Research Triangle Park, NC, 27709, USA

## Abstract

**Background:**

Retrieving pertinent information from biological scientific literature requires cutting-edge text mining methods which may be able to recognize the meaning of the very ambiguous names of biological entities. Aliases of a gene share a common vocabulary in their respective collections of PubMed abstracts. This may be true even when these aliases are not associated with the same subset of documents. This gene-specific vocabulary defines a unique fingerprint that can be used to disclose ambiguous aliases. The present work describes an original method for automatically assessing the ambiguity levels of gene aliases in large gene terminologies based exclusively in the content of their associated literature. The method can deal with the two major problems restricting the usage of current text mining tools: 1) different names associated with the same gene; and 2) one name associated with multiple genes, or even with non-gene entities. Important, this method does not require training examples.

**Results:**

Aliases were considered “ambiguous” when their Jaccard distance to the respective official gene symbol was equal or greater than the smallest distance between the official gene symbol and one of the three internal controls (randomly picked unrelated official gene symbols). Otherwise, they were assigned the status of “synonyms”. We evaluated the coherence of the results by comparing the frequencies of the official gene symbols in the text corpora retrieved with their respective “synonyms” or “ambiguous” aliases. Official gene symbols were mentioned in the abstract collections of 42 % (70/165) of their respective synonyms. No official gene symbol occurred in the abstract collections of any of their respective ambiguous aliases. In overall, querying PubMed with official gene symbols and “synonym” aliases allowed a 3.6-fold increase in the number of unique documents retrieved.

**Conclusions:**

These results confirm that this method is able to distinguish between synonyms and ambiguous gene aliases based exclusively on their vocabulary fingerprint. The approach we describe could be used to enhance the retrieval of relevant literature related to a gene.

## Background

Modern Biological Sciences rely on the worldwide interchange of results published in peer reviewed journals and indexed in a freely accessible database such as PubMed, provided by the (US) National Center for Biotechnology Information (NCBI). PubMed currently (September 2009) contains over 19 million abstracts, and grows at a pace of one more abstract per minute. Retrieving pertinent information from this vast resource requires cutting-edge text mining methods capable of resolving the meaning of ambiguous words which are widely spread in the biological literature. Genome-wide approaches are largely used to search for genes that cause diseases or regulate physiological conditions of interest. These techniques often identify many hundreds of candidate genes. Selection of the most probable of these candidate genes for further empirical analysis requires integration of data-mining of gene expression data and text-mining of biomedical literature. When searching information about genes or proteins in the biomedical literature, two main problems arise. One is to relate information in different documents that refer to the same gene but use different symbols. Querying PubMed using only the official gene symbols will produce only a subset of the actual text corpus associated with that gene and relevant information may be skipped. The other problem, probably more intricate, is to recognize the contextual meaning of single gene symbols that may refer to multiple genes, or may also be the abbreviation of terms with completely different, non-gene meanings [1]. To provide the reader with an idea about the relevance of these two problems, it has been estimated that at least 30% of human genes are affected by homonymy [2]. The challenging task of resolving these ambiguities is further aggravated by the fact that only 30% of the gene symbols in PubMed abstracts are accompanied by a matching long form [3].

A wide variety of approaches have been proposed to assign proper sense to an ambiguous term. In the biological field, supervised machine learning methods have been proposed for disambiguation of gene names/aliases [4,5]. A drawback of these methods is that they require a number of training examples for each of the possible senses. These training sets are often difficult to obtain. For example, Podowski et al [5] used Bayesian classifier models to disambiguate gene symbols found in LocusLink. Their system can distinguish between gene and non-gene meanings of a symbol. In their proof of concept experiment using 66 manually curated gene symbols, they reached an accuracy of 90%, but only when more than 20 abstracts per gene meaning were available for training. Alternatively, Schijvenaars and cols. [2] developed a method which relies on a thesaurus to find biomedical concepts in text containing gene symbols. This strategy consists in “concept fingerprinting” reference documents associated with a given gene name and then comparing the “concept fingerprints” of the reference set with those of documents in the test set.

We present herein an original method for estimating the ambiguity level of individual aliases in large gene terminologies based exclusively in their name-specific vocabulary fingerprints automatically extracted from the literature. These vocabulary fingerprints are extracted from text corpora of PubMed abstracts using a previously published algorithm [6]. The method can deal with the two major problems restricting the usage of current text mining tools: 1) different names associated with the same gene; 2) and one name associated with multiple genes, or even with non-gene meanings. Moreover, this method does not require training examples which is a major advantage compared to supervised approaches.

## Results and Discussion

### The final text corpus

The initial gene terminology comprised 100 EntrezGene official gene symbols and 425 aliases, accounting for 525 cases. The casuistic of the study was meant to reflect the scale of a typical literature or pathway mining exercise in support of a focused gene expression analysis. PubMed abstracts were retrieved for 73 official gene symbols and 256 aliases, forming a text corpus of 13,355 abstracts with 21% redundancy (Table 1). Redundancy may be in part explained by the same document being retrieved with different synonyms of a same gene. However, in some cases the same abstract refers to different genes, suggesting functional associations between them, despite the fact that they had been randomly chosen in this study. The full list of official gene symbols and aliases used in this study is presented in additional file 1: EntrezGene official symbols with PubMed abstracts and its aliases classified by the algorithm, and in additional file 2: EntrezGene official symbols without PubMed abstracts and their aliases.

**Table 1:** Dataset description

	Initial dataset	Dataset with PubMed abstracts	Dataset fulfilling the algorithm’s requirements*	Final dataset (ambiguous aliases excluded)
EntrezGene official symbols	100	73	68**	68
Aliases	425	256	223	165
Abstracts in text corpus	-	13355	12088	9005
Unique PubMed IDs in text corpus	-	11022	10312	7523
Redundancy in text corpus (%)	-	21	16.6	19.7

* The algorithm requires the official gene symbol, and at least one alias and one internal control to produce text corpora of PubMed abstracts. Additionally, the algorithm requires an informative group-specific vocabulary to pass the filters for ubiquitous terms.

** Five official gene symbols, namely DERL3, KCNA7, KCNJ14, MED18, and TBRV4-2, did not fulfil the algorithm’s requirements since their aliases produced no PubMed abstract.

Because we aimed to assess the ambiguity level of a gene alias in the context of its group, the algorithm requires the official gene symbol, at least one alias and at least one internal control to produce text corpora of PubMed abstracts. Additionally, the algorithm requires an informative group-specific vocabulary to pass the filters for ubiquitous terms, as described in the “Methods” section. Twenty-seven genes were automatically excluded because they produced no text corpora, even though 32 of their aliases had abstracts in PubMed (additional file 2: EntrezGene official symbols without PubMed abstracts and their aliases). Other 116 aliases whose official gene symbols had abstracts in PubMed did not produce text corpora when used to query PubMed and were automatically excluded from the analysis, and thus were not classified neither as synonyms, nor as ambiguous. Their exclusion did not affect the analysis of their respective genes, i.e., their official gene symbols and aliases with abstracts (additional file 1). Alias H6 of the HMX1 gene (additional file 1) had PubMed abstracts but was excluded because its informative vocabulary did not pass the filters for ubiquitous terms. HMX1 had one alias classified as synonym: “homeo box (H6 family) 1”. Five genes (DERL3, KCNA7, KCNJ14, MED18, and TBRV4-2) out of 73 (7%) whose official gene symbols produced text corpora were excluded because none of their aliases had PubMed abstracts (additional file 1).

### Fine tuning the algorithm’s parameters

**Figure 1:**
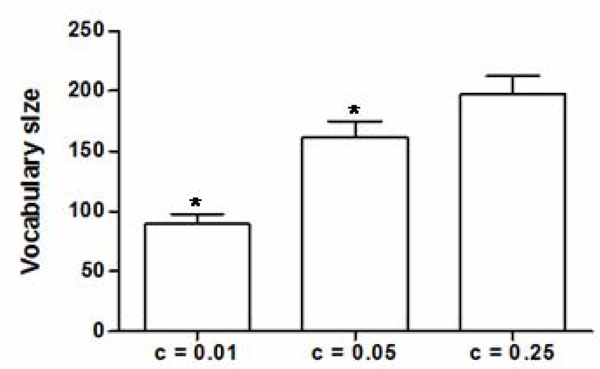
Stringency thresholds and vocabulary size. C = thresholds. * = p < 0.05.

We assessed the effect of increasing the stringency of vocabulary filtering by testing three thresholds of baseline cut-off (“c”), i.e.: 0.25, 0.05, and 0.01. This means that any term which frequency in the baseline abstract collection was equal of greater than 25, 5, or 1%, accordingly to the c value chosen, was automatically excluded from the vocabulary of the group. These terms are considered broadly spread in the unspecific literature and might not be useful to discriminate any specific entity. Increasing the stringency did not influence the number of genes (groups) in the final dataset (68) and had only a minor effect on the number of aliases passing the filters (221, 223, and 224, for c = 0.01, c = 0.05, and c = 0.25 respectively). Increasing the stringency to 0.01 significantly reduced the size of the group-specific vocabularies (Figure 1). However, this effect was not accompanied by any significant increase in the delta of the Jaccard distance between the official gene symbols and their respective aliases, and between the official symbols and the internal controls (data not shown).

### Proof of concept using the baseline cut-off = 0.05

**Figure 2:**
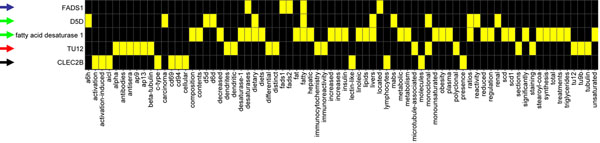
Vocabulary fingerprint for FADS1 and its aliases. Schematic description of a group-specific informative vocabulary automatically extracted from a text corpus of PubMed abstracts. In this example, two “synonyms” (green arrows) and one “ambiguous” alias (red arrow) of official gene symbol FADS1 (which encodes the enzyme fatty acid desaturase 1; blue arrow) are distinguished by the algorithm when baseline cut-off was set at c = 0.05. The internal control is the unrelated official gene symbol CLEC2B (black arrow). The Jaccard distances to FADS1 are: 1) D5D = 0.937; 2) fatty acid desaturase 1 = 0.944; 3) TU12 = 1; CLEC2B = 1. Yellow boxes = words from the group-specific informative vocabulary that occur in the text corpora of a given gene symbol or alias.

The average number of aliases per gene in the final dataset was 3.3 (223 aliases / 68 genes). Jaccard distances were calculated between the official gene symbol and its aliases or unrelated internal controls in the group in order to enable the determination of the ambiguity of a symbol. In this proof of concept study, we classified an alias as “ambiguous” when its Jaccard distance to its respective official gene symbol was equal of greater than the Jaccard distance between its official symbol and any internal control (exemplified in Figure 2). Using this threshold we were able to disclose 58 “ambiguous” aliases for 36 genes (1.6 ambiguous alias / gene). After excluding the “ambiguous” aliases, the average number of synonyms per gene in the dataset shrunk to 2.4 (165 aliases / 68 genes). (see additional file 1: EntrezGene official symbols with PubMed abstracts and its aliases classified by the algorithm, and additional file 3: Jaccard distances between the EntrezGene official symbols and their respective aliases).

The average number of abstracts retrieved per official gene symbol was 30.9 (2099/68). Combining the abstracts obtained querying PubMed with official gene symbols and their respective aliases classified as “synonyms” increased the average size of text corpora to 132 (9005/68) abstracts / gene. Redundancy accounted for only 19.7 % of the global text corpus (7523 unique PubMed IDs) (Table 1). In overall, querying PubMed with official gene symbols and “synonym” aliases allowed a 3.6fold increase in the number of unique documents retrieved.

We estimated the algorithm’s performance by measuring the frequencies of official gene symbols in the abstract collections retrieved querying PubMed with their respective aliases classified as “synonym” or “ambiguous”. Official gene symbols occurred in 40.6 % (67/165) of the abstract collections retrieved with their respective “synonyms”. No official gene symbol was mentioned in any abstract collection retrieved with its respective “ambiguous” aliases. These results confirm that the present method is able to distinguish between unambiguous and ambiguous gene aliases based exclusively on the vocabulary present in their associated literature. Acknowledging official gene symbols are not necessarily mentioned in the literature of their respective gene aliases, the percentages above should not be mistaken as a measure of the sensitivity / specificity of the method.

### Two case studies to assess the enrichment in relevant information about a gene obtained by querying PubMed with “synonym” aliases

Querying PubMed with the official symbol FADS1 produced a text corpus comprising 28 abstracts all related to this gene (PMIDs: 10860662; 15168598; 16367923; 16670158; 16893529; 17786358; 17823443; 18030445; 18155511; 18320251; 18479586; 18626191; 18652865; 18671863; 18763007; 18842780; 18936223; 19043545; 19060906; 19060910; 19091074; 19148276; 19195843; 19443042; 19573581; 19689798; 19752397; 19776639). In this text corpus we found that the delta-5 desaturase, encoded by FADS1, is a rate-limiting enzyme in the desaturation of linoleic acid to arachidonic acid [7,8], which is incorporated in phospholipids and is a precursor of molecules involved in inflammation and immune response. Indeed, fatty acid composition of serum phospholipids is genetically controlled by the FADS1 FADS2 gene cluster [9,10]. Hepatic desaturase activities have been implicated in insulin resistance, obesity and dyslipidaemia [11]. Nevertheless, FADS1 is differentially expressed in hepatocellular carcinoma [12] and its methylation has been reported in primary gastric cancer [13].

When the alias D5D, classified as "synonym" by our method, is used to query PubMed, 23 abstracts were retrieved (PMIDs: 2585642; 3821914; 3891589; 3904980; 4023915; 9976912; 11414679; 11686594; 11792729; 12440976; 15740094; 15782269; 16132958; 16734456; 16988497; 17307914; 17639524; 17852835; 18030445; 19060426; 19228394; 19340699; 19712485). Only one abstract (PMID 18030445) was indexed in PubMed to both the official gene symbol FADS1 and the “synonym” alias D5D. Ten out of these 23 abstracts were not related to FADS1 (PMIDs 2585642; 3821914; 3891589; 3904980; 4023915; 9976912; 11686594; 15740094; 15782269; 16734456; 17639524). However, in the remaining abstracts indexed only to D5D we found that: a) D5D expression is dual regulated by SREBP-1c and PPARalpha in mice [14]; b) and by dietary vitamin A and exogenous retinoic acid in liver of adult rats [15]; c) the ratio of administered n6 to n3 fatty acids regulates the transcription of FADS1 in human hepatocytes [16]; d) in HL60 cells, a promyelocytic cell line resembling human leukocytes, when the supply of fatty acids is limited, the intracellular content of n3 and n6 fatty acids decreases and this leads to up regulation of D5D [17]; e) intake of high saturated fatty acids and monosaturated fatty acids appears to increase expression of D5D in peripheral blood mononuclear cells, whilst essential fatty acids intake appears to decrease expression of D5D [18]; f) low D5D activity is associated with metabolic syndrome independent of lifestyle factors such as smoking, physical activity, etc [19,20]; g) peroxisome proliferators (PPs), besides increasing fatty acid degradation, induces FADS1 as a compensatory response to an increased demand for unsaturated fatty acids [21].

Eight abstracts in PubMed were indexed to the "synonym" alias "fatty acid desaturase 1" (PMIDs: 10860662; 15168598; 16893529; 17176482; 17761144; 18222430; 18479586; 19573581); five out of them were indexed to both FADS1 and the "synonym" alias "fatty acid desaturase 1" (PMIDs: 10860662; 15168598; 16893529; 18479586; 19573581). Among the remaining three abstracts indexed only to “fatty acid desaturase 1”, the first one reported decreased expression levels of “fatty acid desaturase 1” in women under treatment with estrogen [22]. The second abstract reported “fatty acid desaturase 1” up regulation in dystrophic muscles of patients with limb-girdle muscular dystrophy [23]. The third abstract reported that fatty acid desaturase 1 from *Capsicum annum* (red pepper) may play a role in hypersensitivity response induced by infection with tabacco mosaic virus. The authors demonstrated that suppression of “fatty acid desaturase 1” caused blocking of cell death induced by Bcl2-associated X (Bax) protein in tabacco plants [24].

Finally, three abstracts (PMIDs: 1717594; 3835499; 6699682) were indexed in PubMed to the alias TU12 classified as “ambiguous” by our method; none of them are related to FADS1. It became clear from these findings that TU12 had been erroneously assigned as an alias of FADS1 in the GATE terminology used in this study.

LLCDL1 and FADSD5, two aliases of FADS1 which were present in the initial gene name list (additional file 1: EntrezGene official symbols with PubMed abstracts and their aliases classified by the algorithm) extracted from GATE had no abstracts in PubMed and thus, could not be classified by the algorithm which is based on literature profiling.

Thus, querying PubMed with the official gene symbol FADS1 and its aliases classified as “synonyms” by our method lead to a 1,5 fold increase in the number of unique and relevant abstracts retrieved compared to the situation when only the official gene symbol is used to compose the query (from 28 to 43 abstracts). Important information was found only in the additional abstracts indexed to the “synonym” aliases but not to the official gene symbol. On the contrary, the “ambiguous” alias produced only documents that were not related to FADS1.

In a second case study, the gene ADD2, encoding the adducing 2 isoform a, was analyzed. Adducins are cytoskeletal actin-binding proteins and take part in the junctional complexes. Among their various know functions, they are constituents of synaptic structure [25] and play a role in cerebrospinal fluid homeostasis [26]. Adducins alpha and beta (ADD2) have been implicated in hypertension [27] and renal dysfunction [28]. When the official gene symbol (ADD2) was used to query PubMed, 26 abstracts with pertinent information were retrieved (PMIDs: 7490111; 9012501; 9244430; 10485892; 11082136; 12951058; 15474463; 15528469; 15699449; 15716695; 15928065; 15963851; 16497648; 16565244; 16604465; 17301826; 17465710; 17854487; 18003638; 18482449; 18667944; 18723693; 18787518; 18959617; 19838659; 19900187). Curiously, one abstract (PMID: 9012501) indexed by PubMed to ADD2 refers to arrested development (add) in *Arabdopsis*. Querying PubMed with the synonym alias “adducing 2 isoform a” produced a text corpora with 16 abstracts (PMIDs: 1556101; 2524283; 7864813; 8239658; 8952067; 8913030; 9244430; 9354614; 9524222; 11598638; 12675919; 12969891; 15329129; 17610345; 18344231; 18757509) from which only one was not pertinent (PMID: 18757509); another abstract (PMID: 9244430) was also present in the text corpus of the official gene symbol ADD2. This means an information increment of ~1.6 fold when using the official gene symbol and the synonym alias to query PubMed. The ambiguous alias ADDB had 25 abstracts in PubMed (PMIDs: 1646786; 1905712; 2177138; 7565602; 7746142; 8387145; 8510642; 8752329; 8885269; 9004227; 9023205; 9781875; 11292820; 11544244; 11810266; 15066813; 15099822; 16385024; 16780573; 17499012; 18573180; 19129187; 19395381; 19542287; 19620647), but none were related to ADD2.

## Conclusions

The method presented herein estimates the ambiguity level of gene aliases based on their vocabulary fingerprint extracted from their respective abstract corpora downloaded from PubMed. This original approach does not require training sets of manually curated documents and only needs to be loaded once to a whole gene terminology. No information is lost because, since the aliases have been scored, customized cut-off thresholds can be applied to specific aims. The method could be used in the generation of more powerful queries to retrieve literature of significance to the study of a particular gene.

## Methods

### Building the gene terminology and the text corpora

A gene terminology containing 100 human genes was randomly picked from GSK’s database (Genes And Targets Explorer). GATE combines data from different internal and external sources, including **EntrezGene** [http://www.ncbi.nlm.nih.gov/entrez/query.fcgi?db=gene]. A restriction was imposed so that selected genes had at least four aliases. The full list of EntrezGene official gene symbols and aliases used in this pilot is presented in additional file 1: “EntrezGene official symbols with PubMed abstracts and its aliases classified by the algorithm”, and in additional file 2: “EntrezGene official symbols without PubMed abstracts and their aliases”. Up to 100 most recent abstracts were obtained for each official gene symbol or alias (cases) by automatically querying **PubMed** [http://eutils.ncbi.nlm.nih.gov/]. These related cases were treated as a group. Sets of abstracts retrieved with up to three unrelated randomly picked official gene symbols (some official symbols did not produce abstract collections) were added to each group as internal controls. The unrelated gene names were obtained by shuffling the list of official symbols in the initial gene terminology.

### Implementing the algorithm

We implemented a modified version of the algorithm of Chaussabel & Sher [6] in a Perl programme. This algorithm extracts the informative vocabulary from the text corpus containing all abstracts retrieved for all official gene symbols and aliases being compared in each group. Then, it calculates, for each official gene symbol or alias, the fraction of its specific abstract collection containing each term found in the informative vocabulary of the group. Finally, each official gene symbol and aliases are represented as a vector of terms and their relative frequencies. For our purposes, a term is defined as any string of at least 3 alphanumeric characters (numeric strings were discarded). The occurrences of singular and plural forms of the same term were combined using the **Damian Conway's Perl module Lingua::EN::Inflect version 1.89** [http://www.csse.monash.edu.au/~damian/CPAN/Lingua-EN-Inflect.tar.gz]. To reduce dimensionality of vectors eliminating ubiquitous terms and selecting only those that can be found in most abstracts of gene-specific collections and show a low baseline occurrence in the general literature, a set of filters are successively applied to the raw data. First, the algorithm determines the baseline frequencies of each term in a set of 7465 PubMed abstracts retrieved for a set of 230 official gene symbols randomly picked. Terms with frequency higher than the cut-off baseline are eliminated from the vocabulary of the experimental set of genes.

The best cut-off baseline (“c” as described in [6]) was determined empirically by comparing the results obtained with different values, i.e.: 1, 5, and 25%. For the remaining vocabulary passing this first filter, the difference cut-off between term occurrence in the experimental set and its baseline occurrence is optimized applying the following equation: *cut-off = t + (k/n)* where *t* is the minimum threshold, *k* is a constant and *n* is the number of abstracts retrieved for a given official gene symbol or alias. For gene names with five or less abstracts, *n* was set at five. This equation partially compensates the difference in the number of abstracts retrieved for each gene name. In the present study, we used *t* = 0.15 and *k* = 1.5 as set in the original paper describing the algorithm [6].

A term-by-gene matrix of term-frequencies is generated to each group. Frequencies are then converted to discrete values (0 or 1) and used to calculate the Jaccard distance between the official gene symbol and each of its aliases and between official gene symbol and the internal controls.

Jaccard distance = 1 – (n11 / n11 + n01 + n10)

where n11 is the number of terms occurring in the name-specific vocabularies of the official symbol and the alias (or internal control); n10 is the number of terms occurring in the name-specific vocabulary of the official symbol, but not in the name-specific vocabulary of the alias (or the internal control); n01 is the number of terms occurring in the name-specific vocabulary of the alias (or internal control), but not in the name-specific vocabulary of the official symbol. Jaccard distance values range from 0 (perfect match) to 1 (no match).

### Statistics

Data were analysed with the Kruskall-Walllis test and differences between groups were tested by Dunn’s Multiple Comparison test. A value of p < 0.05 was considered significant. The Kruskal-Wallis test compares more than two unpaired groups that did not form a normal distribution. Dunn's post test calculates a P value for each pair of columns. The calculation of the P value takes into account the number of comparisons made. Dunn’s post test is based on the assumption that the probability of occurrence of one or more events can never exceed the sum of their individual probabilities [29].

### Assessing the algorithm’s performance

We evaluated the coherence of the results by determining the frequencies of the official gene symbols in the text corpora retrieved with their respective “synonyms” or “ambiguous” aliases. For this purpose, we used the QDA Miner/WordStat package (Provalis Research, Montreal, Canada).

## Competing interests

The authors declare that they have no competing interests.

## Author’s contributions

RSC conceived of, designed and carried out the study, and drafted the manuscript. DEV and GO participated in the study’s design and data analysis, and helped to draft the manuscript. All authors read and approved the final manuscript.

## Availability and requirements

Project name: Gene aliases disambiguation

Project home page: http://bioinformatics.org/genealiases

FTP site: http://ftp.bioinformatics.org/pub/genealiases/

Operating system: Unix

Programming language: Perl

License: GNU General Public License

Any restriction to use by non-academics: license needed

The authors declare that they have no competing interests.

## Supplementary Material

Additional file 1 **EntrezGene official symbols with PubMed abstracts and their aliases classified by the algorithm.** Description of data: 73 randomly chosen official gene symbols that produced text corpora of PubMed abstracts and their aliases. Aliases were classified by the algorithm as “synonyms”, “ambiguous”, “aliases with PubMed abstract but not passing the filters”, or “aliases without PubMed abstracts”. Click here for file

Additional file 2 **EntrezGene official symbols without PubMed abstracts and their aliases.** 27 randomly chosen official gene symbols that did not produce text corpora of PubMed abstracts, and their aliases. Aliases were classified as “aliases with PubMed abstract but not passing the filters”, or “aliases without PubMed abstracts”. Click here for file

Additional file 3 **Jaccard distances between the official gene symbols and their respective aliases.** For 36 genes the distance between the official gene symbol and at least one of its aliases (red circles) exceeded the distance between the official symbol and the internal control (black circles). Green circles represent the distance between the official gene symbol and aliases classified as “synonyms”. Click here for file
