# Supplementary figures and images for: Disclosing ambiguous gene aliases by automatic literature profiling
Source: BMC Genomics. 2010 Dec 22;11(Suppl 5):S3. doi: 10.1186/1471-2164-11-S5-S3 (PMC3045796; doi:10.1186/1471-2164-11-S5-S3)

Jaccard distance

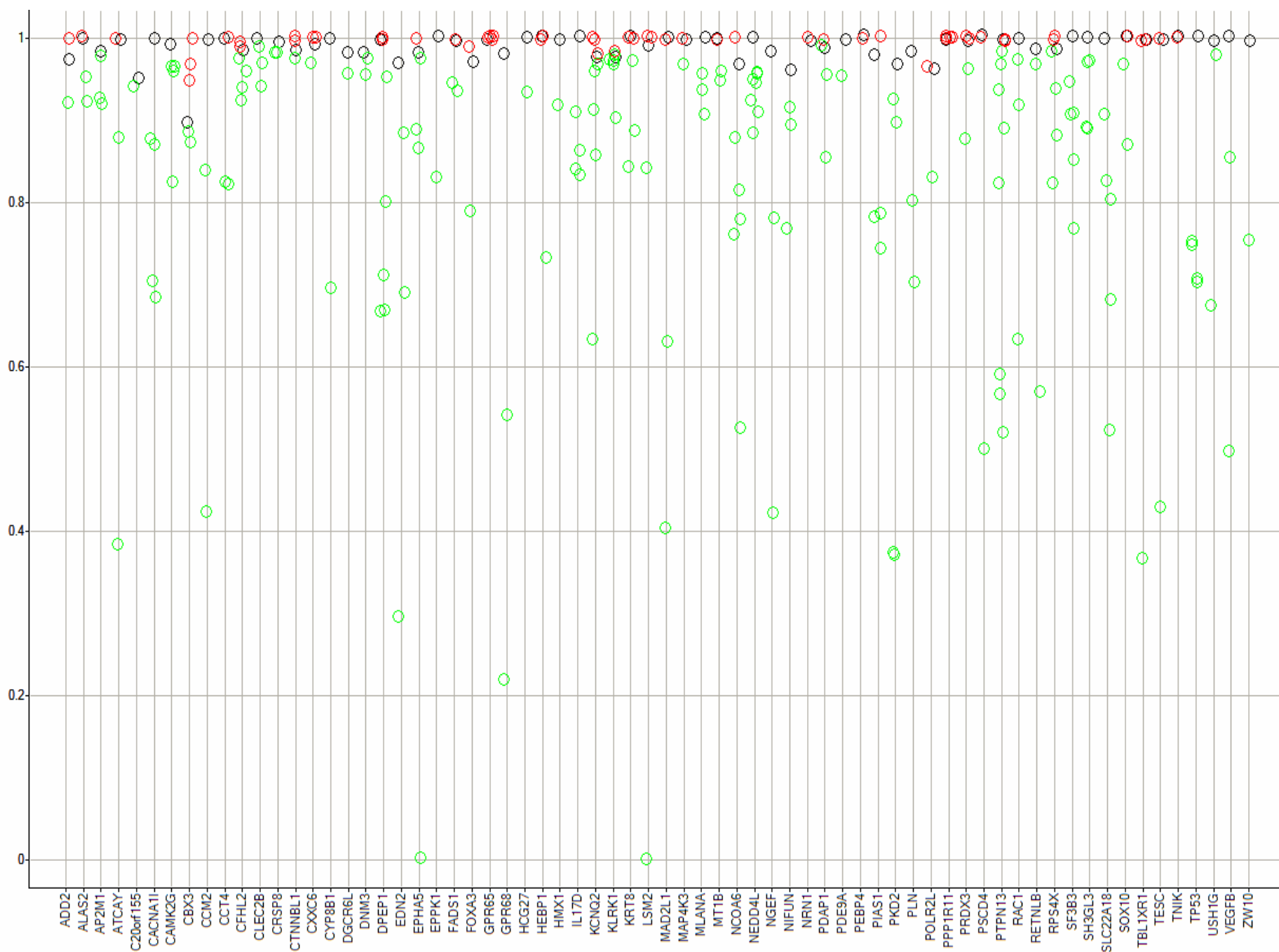

EntrezGene official symbols

Supplement: Additional file 3 — Jaccard distances between the official gene symbols and their respective aliases. For 36 genes the distance between the official gene symbol and at least one of its aliases (red circles) exceeded the distance between the official symbol and the internal control (black circles). Green circles represent the distance between the official gene symbol and aliases classified as “synonyms”. [file 1471-2164-11-S5-S3-S3.pdf]
